# Copper metabolism and hepatocellular carcinoma: current insights

**DOI:** 10.3389/fonc.2023.1186659

**Published:** 2023-07-05

**Authors:** Cheng Zhou, Jinqiu Yang, Tong Liu, Ran Jia, Lin Yang, Pengfei Sun, Wenxia Zhao

**Affiliations:** ^1^ The First College of Clinical Medicine, Henan University of Chinese Medicine, Zhengzhou, China; ^2^ Department of Hepatobiliary Surgery, Xianyang Central Hospital Affiliated to Shaanxi University of Chinese Medicine, Xianyang, China; ^3^ Department of Orthopaedics, Jiangsu Province Hospital of Chinese Medicine, Nanjing, China

**Keywords:** copper, hepatocellular carcinoma, biology, treatment, molecular mechanism, cancer

## Abstract

Copper is an essential trace element that acts as a cofactor in various enzyme active sites in the human body. It participates in numerous life activities, including lipid metabolism, energy metabolism, and neurotransmitter synthesis. The proposal of “Cuproptosis” has made copper metabolism-related pathways a research hotspot in the field of tumor therapy, which has attracted great attention. This review discusses the biological processes of copper uptake, transport, and storage in human cells. It highlights the mechanisms by which copper metabolism affects hepatocellular carcinogenesis and metastasis, including autophagy, apoptosis, vascular invasion, cuproptosis, and ferroptosis. Additionally, it summarizes the current clinical applications of copper metabolism-related drugs in antitumor therapy.

## 1 Introduction

Primary liver cancer is a prevalent malignant tumor that ranks as the third most common cause of cancer-related deaths worldwide, and is particularly frequent in East Asia and Southeast Asia (1). Hepatocellular carcinoma (HCC) is the most common type of primary liver cancer, accounting for 75%-85% of cases. Finding new breakthroughs in treatment has been the key to research on HCC, as HCC has a 5-year survival of less than 15% (2). Copper is a widespread metallic element in nature. With the interdisciplinary development, the pathways of copper metabolism in human body are gradually discovered. The redox properties of copper are both beneficial and potentially toxic to cells, which are increasingly found to be involved in cell proliferation and death pathways in a rising number of studies (3). The metallic signal of copper is strongly associated with tumor progression, especially HCC, where the liver is the main organ for copper storage (4). Key enzymes and genes related to copper metabolism have become important directions in the treatment of HCC, while the exploration of copper in the diagnosis, treatment, prognosis and survival analysis of HCC has become a current research topic.

This review aims to provide an overview of the normal physiological metabolic processes of copper, a summary of the mechanisms of copper metabolism involved in the progression of HCC, and a discussion of recent research advances in the treatment of HCC through the regulation of copper homeostasis.

## 2 Copper metabolic process in human body

### 2.1 Copper absorption

Copper, which is an essential cofactor, is widely found in living organisms in nature. Diets high in copper, such as offal, shellfish, seeds, legumes, vegetables and whole grain cereals, are the main ways in which the body obtains copper, and industrial products may also be an important source of copper in the human body (5). The intestinal epithelium is responsible for the absorption of dietary copper, which is removed through the liver if the level is high in the body and secreted by the bile into the gastrointestinal tract and excreted in the feces. To keep free copper at a low level, copper ions in the body mainly attach to certain proteins or other molecules to ensure normal biochemical processes (6). Since its unstable redox potential, copper homeostasis is essential for cell survival.

Copper is absorbed in the small intestine, with the duodenum serving as its primary absorption site and having an absorption efficiency of up to 60% (7). Copper in the diet is usually present in the form of Cu^2+^, but only Cu^+^ can be absorbed and reused. This process is mainly involved by the prostate metal reductase six transmembrane epithelial antigen of the prostate (STEAP) and duodenal cytochrome b (DCYTB) (8, 9). Cu^+^ is taken up into the cell mediated by copper transporters 1 (CTR1) on the apical side of the enterocyte. Cells can also take minor amounts of Cu^2+^ up, but the underlying mechanism is not clear. The low affinity copper transporter receptor 2 (CTR2), divalent metal transporter1 (DMT1), and sodium-dependent amino-acid transporters may explain this mechanism as alternative copper uptake pathways (10).

### 2.2 Copper transportation

There are two membrane-bound copper-transporting adenosine triphosphatases (ATPases) exist in human cells, ATP7A and ATP7B, both of which play an important role in the digestive tract. ATP7A may promote the efflux of Cu^+^ from the intestinal epithelium and transport to the circulation (11, 12). ATP7B is primarily responsible for the storage of Cu^+^ in intracellular vesicles to maintain the copper balance required for normal homeostasis of the intestinal epithelial (13).

Ceruloplasmin (CP) is the main carrier involved in copper transport, and each CP can bind six Cu^+^ (14). In addition, albumin and histidine are also involved in the transport of copper. Copper is secreted by intestinal epithelial cells into the portal vein circulation and bound with these copper-carrying protein. When Cu^+^ is transported to the liver, they would be mediated by CTR1 into hepatocytes. In the cytoplasm, Cu^+^ would be isolated by glutathione (GSH) and stored in metallothioneins (MTs) (11). Both have a high affinity for copper and are rich in thiol groups, which helps to maintain a low quantity of free copper. Usually, liver is one of the main organs storing Cu^+^, with MT1 and MT2 being the main storage sites for Cu^+^. But a new study shows that MT3, which is highly expressed in the central nervous system, is one of the major players in copper homeostasis (15).

In addition, a portion of the Cu^+^ will bind to the copper chaperones and transported to specific organelles to participate in related physiological process. In mitochondria, Cu^+^ is involved in the respiratory chain and redox pathways by binding to cytochrome c oxidase (CCO). For example, the copper chaperones (COX17, COX19 and COX23) are responsible for transporting Cu^+^ to the mitochondria, and then which is delivered to CCO by the mitochondrial inner membrane proteins Sco1, Sco2 and COX11.

Antioxidant protein 1 (ATOX1) would transport Cu^+^ to the Trans-Golgi Network (TGN) and promote the synthesis of copper enzymes such as lysyl oxidase, tyrosinase and copper cyanobactin (16). Except for the liver, ATP7A are expressed in most tissues (16). However, ATP7B are only present in hepatocytes, which pump Cu^+^ from the cytoplasm into the TGN. When excess copper enters the hepatocyte, endolysosomal vesicles containing ATP7B would transport them to bile duct and drain the excess Cu^+^ into the bile (17). Therefore, mutations in ATP7A and ATP7B predispose to disorders of copper metabolism, allowing Cu^+^ to accumulate in cells, which leads to the onset of Menkes’ disease and Wilson disease (18).

**Figure 1:**
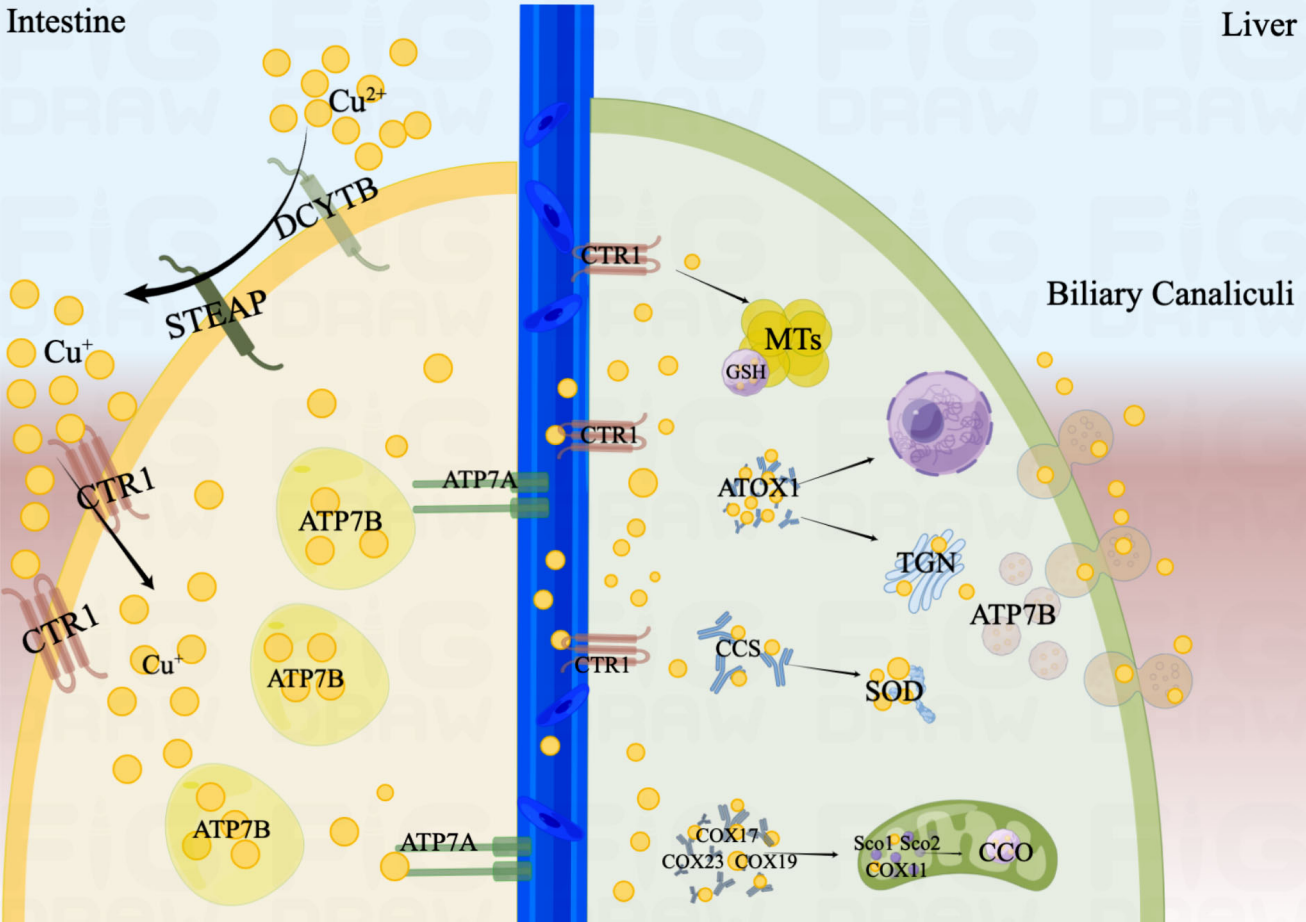
The process of copper absorption and transport in the human body. Cu^2+^ is restored to Cu^+^ by STEAP and DCYTB in the small intestine. Cu^+^ is taken up into the cell mediated by CTR1. ATP7B is primarily responsible for the storage of Cu^+^, and ATP7A may promote the efflux of Cu^+^ from the intestinal epithelium and transport to the circulation. When Cu^+^ is transported to the liver, they would be mediated by CTR1 into hepatocytes. Cu^+^ would be isolated by GSH and stored in MTs. In mitochondria, Cu^+^ is involved in the respiratory chain and redox pathways by binding to CCO. ATOX1 would transport Cu^+^ to the TGN and promote the synthesis of copper enzymes. When excess copper enters the hepatocyte, endolysosomal vesicles containing ATP7B would excrete Cu^+^ into the bile. In addition, CCS would also transport Cu^+^ to SOD to alleviate oxidative stress. In the nucleus, Cu^+^ can be combined with transcription factors and drive gene expression.

In addition, Copper Chaperone (CCS) would also transport Cu^+^ to superoxide dismutase (SOD) to alleviate oxidative stress and maintain copper homeostasis (19, 20). In the nucleus, Cu^+^ can be combined with transcription factors and drive gene expression (21). The process of copper absorption and transport in the human body is shown in **Figure 1**.

## 3 Association between copper metabolism and HCC

### 3.1 Strong association between high copper level and the prevalence of HCC

The role of copper in biological processes has been a hot topic of research for this century. Numerous factors regulate and maintain the body’s intake, transport, and secretion of copper in a dynamic equilibrium. Aberrant copper metabolism or copper-induced cell death can lead to a variety of diseases when copper homeostasis is disrupted in the body. Low level of Cu can impair the function of metal-binding enzymes, while too high level can lead to abnormal cellular functions (4). A number of studies have shown that tumor tissues require higher level of copper to meet the high metabolic demands compared to healthy tissues (22). Elevated copper levels have now been found to be associated with a multitude of malignancies according to research, including breast cancer (23), colorectal cancer (24), lung cancer (25), and gallbladder cancer (26). Copper ions were absorbed through the intestine, arriving in the liver from the portal vein with serum proteins as carriers, and enter the hepatocytes via CTR1, where large amount of copper was stored in the hepatocytes in combination with MT1 and MT2 (27). The liver is the center of Cu storage and transport as a core player in the regulation of systemic Cu homeostasis. The liver cells will be the first to be impacted when there is an abnormality in the copper metabolism. Therefore, Cu is closely associated with the development of liver disease. Wilson disease, as we know it, has a defect in copper processing resulting in high copper level and toxic effects on liver cells (28). Mitochondrial damage induced by copper overload results in other liver lesions in more than half of Wilson disease patients (29). Cirrhosis, the end-stage of many liver diseases, is an essential risk factor for HCC, which also exhibits abnormal accumulation of copper and abnormal distribution of other trace metal elements (30). Both European and Asian cohort studies have shown that elevated copper levels in humans are associated with a high risk of morbidity and a poor prognosis of HCC (31, 32). Cuproptosis-related genes have potential for constructing a prognostic model for HCC (33).

### 3.2 Disorders of copper metabolism play a major role in the development of hepatocellular carcinoma

#### 3.2.1 Autophagy

**Figure 2:**
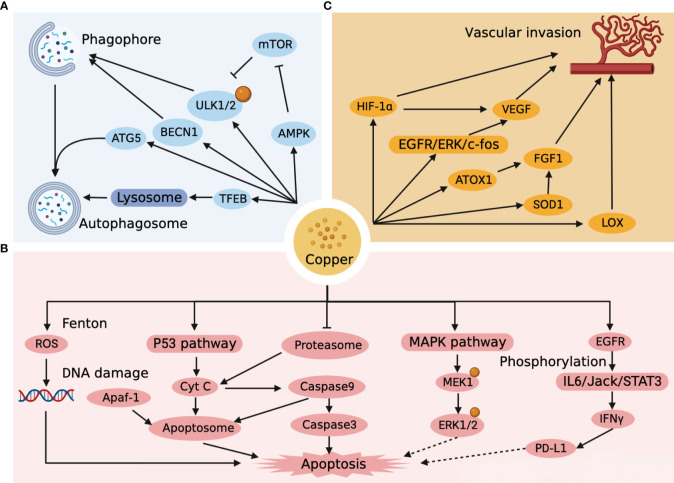
Targets and pathways of autophagy, apoptosis and vascular invasion associated with copper metabolism in HCC. **(A)** Copper impacts the autophagic pathway of HCC by affecting the formation of phagophore, autophagosome, and lysosome. **(B)** Copper directly affects the apoptotic pathway of HCC by generating ROS, forming apoptosome, and activating the apoptotic cascade response, and indirectly affects the apoptotic pathway of HCC by regulating ERK1/2 and PD-L1. **(C)** Copper affects vascular invasion by affecting endothelial cells as well as HCC metastasis.

Autophagy is a highly regulated cellular mechanism that aimed at bioenergetic recovery through intracellular destruction and breakdown of dysfunctional cytoplasmic components and recycling of energy (34). Autophagy is a double-edged sword. On the one hand, autophagy can suppress tumors by reducing reactive oxygen species (ROS) and removing damaged organelles and toxic substances from cells. On the other hand, autophagy inhibits tumor cell apoptosis and provides metabolic support to accelerate the growth of HCC cells. High level of Cu^+^ generates large amounts of ROS during oxidation, which is important for the onset of autophagy (35). Unc-51-like kinase 1 (ULK1/2) is an important initiator of autophagy and is involved in the formation of autophagic vesicles as well as the regulation of the autophagic process. Tsang et al. (36) found that ULK1/2 had a strong affinity for copper. Copper-dependent ULK1/2 activation stimulated autophagic flux, and low intracellular copper (CTR1 depletion, ATP7A overexpression, or copper chelator use) resulted in a decrease of autophagy. In addition, excess copper can also increase autophagic flux by activating the expression of autophagy-related gene 5 (ATG5) (37), Beclin-1 (BECN1) (37)and AMPK-mTOR (38) pathways, as well as accelerate autophagic vesicle formation by mediating TFEB (39) transcription factors. The autophagic targets and pathways associated with copper metabolism in HCC are shown in **Figure 2A**. It is notable that the accumulation of copper and the activation of autophagy in Wilson disease as well as in HCC occur simultaneously (40–42).

#### 3.2.2 Apoptosis

Apoptosis is a programmed death process that is essential in normal development, tissue repair and immune modulation in the human body (43). The Fenton reaction is one of the most common reactions for copper-catalyzed ROS production, whereby high concentrations of copper-induced ROS lead to mitochondrial dysfunction with accelerated apoptosis (44). Moreover, the oxidation of hydroxyl radicals generated by the Fenton reaction can break down the DNA helix structure, resulting in damage to DNA (45). Mitra et al. (46) found that copper-treated spleen and thymus produced different apoptotic pathways. Recent studies (47) have revealed that mitogen-activated protein kinase kinase 1 (MEK1) in the MAPK-ERK pathway also has a high-affinity copper binding site. High concentrations of Cu^+^ stimulate MEK1-dependent phosphorylation of ERK1/2, and the use of copper chelators can target MEK and inhibit tumor proliferation (48). Excessive copper production in the liver induces ROS and mitochondrial transmembrane potential changes that activate the intrinsic pathway of p53 cell apoptosis (49). With the activation of P53, cytochrome C (Cyt C) induces caspase9 to initiate the cascade activation of downstream caspase3, which triggers apoptosis (50, 51). Cyt C, Apoptotic protease-activating factor 1 (Apaf-1) and caspase 9 could also combine to form an “apoptosome”, an apoptotic pathway that induces hepatocyte death (50, 52). The proteasome is a multi-enzyme complex consisting of a 20S core and two 19S regulatory particles (53). It has been shown that proteasome can induce Cyt C into cytoplasm and activate caspase cascade reaction to induce apoptosis, while copper complexes show strong inhibitory ability against proteasome, which may become a new target for tumor therapy (54, 55). It is worth noting that Cu activates the IL6/Jack/STAT3 signaling pathway after catalytic EGFR phosphorylation and is involved in the induction of IFNγ-mediated upregulation of PD-L1 expression (56). An overload of Cu leads to immunosuppression, which not only leads to the development of HCC, but is an important issue in the treatment of HCC. The apoptotic targets and pathways associated with copper metabolism in HCC are shown in **Figure 2B**.

#### 3.2.3 Vascular invasion

Vascular invasion is an important mode of tumor progression as well as metastasis. Notably, copper is a key point in the angiogenic signaling cascade, and copper overload stimulates tumor neovascular growth and invasion, while copper deficiency hinders neointimal formation (57). It was found that copper could activate the EGFR/ERK/c-fos transduction pathway to induce vascular endothelial growth factor (VEGF) expression in hepatoma cells to promote tumor angiogenesis (58). In a further way, copper can also promote the synthesis of FGF-1 through ATOX1 and superoxide dismutase 1 (SOD1) to affect vascular endothelial function, while increasing the invasive and metastatic capacity of tumor cells by activating lysyl oxidase (LOX) (59–61). Copper can even induce the expression of HIF-1α to promote rapid adaptation of tumor cells to hypoxic conditions when the microenvironment is not suitable for tumor growth (62). Copper has a significant facilitating impact in HCC metastasis and angiogenesis, and copper chelators can be used as targeted agents to limit HCC vascular invasion (63). The targets and pathways of vascular invasion associated with copper metabolism in HCC are shown in **Figure 2C**.

#### 3.2.4 Cuproptosis

As early as the 1980s, Halliwell et al. discovered that copper could lead to cell death (64). The mechanism and specific form of copper-induced cell death has long been obscure. In the past, copper ion carriers including disulfiram (DSF) and elesclomol (ES) were considered to have the ability to cause cell death. ES-Cu can bind to ferredoxin 1 (FDX1) and lead to inhibition of Fe-S clusters or act directly on mitochondrial membranes (65), which leads to polarization of the mitochondrial membrane potential and cell death via a ROS-mediated mechanism (66). Similarly, DSF/Cu was also considered to act on the mitochondrial respiratory chain, leading to elevated ROS levels (67). It has only recently been elucidated that cell death induced by copper ion carriers is a copper-induced cell death, independent of the known patterns of cell death. Cuproptosis is a regulated mode of cell death that is distinct from apoptosis and ferroptosis (68). The accumulation of intracellular copper ions acts on the lipid acylation of proteins in the tricarboxylic acid cycle (TCA), triggering proteotoxic stress and inducing cell death (69). Ferredoxin 1 (FDX1), an upstream regulator of protein lipid acylation, has been found to be a key regulator of cuproptosis with significant association with HCC staging and prognosis (70). In contrast to the general population, HCC patients have a much lower level of FDX1 expression, while the low expression of FDX1 suggests a poor prognosis, tumor cells acquire a survival advantage over healthy cells by resisting cuproptosis. In addition, it was found (71) that the cuproptosis-related gene LIPT1 may promote the proliferation and metastasis of HCC, which is a new potential therapeutic target for HCC. It is worth noting that cuproptosis may be an elemental factor in the development of HCC, but experimental studies on cuproptosis are still in their infancy, and most studies have only demonstrated the association of cuproptosis with the prognosis of HCC based on bioinformatic analysis of public databases such as TCGA, ICGC and GEO, so more studies are needed to support this point of view (72–76). The functions of cuproptosis characteristic genes in HCC are shown in **Table 1**.

**Table 1:** Functions of validated cuproptosis genes in HCC.

Gene	Full name	Subcellular locations	Function	Ref
FDX1	Ferredoxin 1	Mitochondrion matrix	Locates upstream of lipoic acid pathway, reducing Cu^2+^ to Cu^+^	(70, 77, 78)
LIPT1	Lipoyltransferase 1	Mitochondrion	Modulation of the lipoic acid pathway, involved in the lipid acylation of DLAT	(71)
CDKN2A	Cyclin-dependent kinase inhibitor 2A	Nucleus and Cytosol	Induces cell cycle arrest in G1 and G2 phases, its mutation is the common molecular anomalies in HCC	(79, 80)
GLS	Glutaminase kidney isoform	Mitochondrion Cytoplasm and cytosol	Catalyzes the catabolism of glutamine	(81–84)
DLAT	Dihydrolipoamide acetyltransferase	Mitochondrion matrix	Mediates the conversion of pyruvate to acetyl-CoA, involved in glycolysis	(85)
PDHB	pyruvate dehydrogenase Β	Mitochondrion matrix	Mediates the conversion of pyruvate to acetyl-CoA, induced metabolic reprogramming of TCA cycle	(86)
MTF1	Metal regulatory transcription factor 1	Nucleus and Cytoplasm	Zinc-dependent transcriptional regulator for metal ions adaption	(87)

#### 3.2.5 Ferroptosis

**Figure 3:**
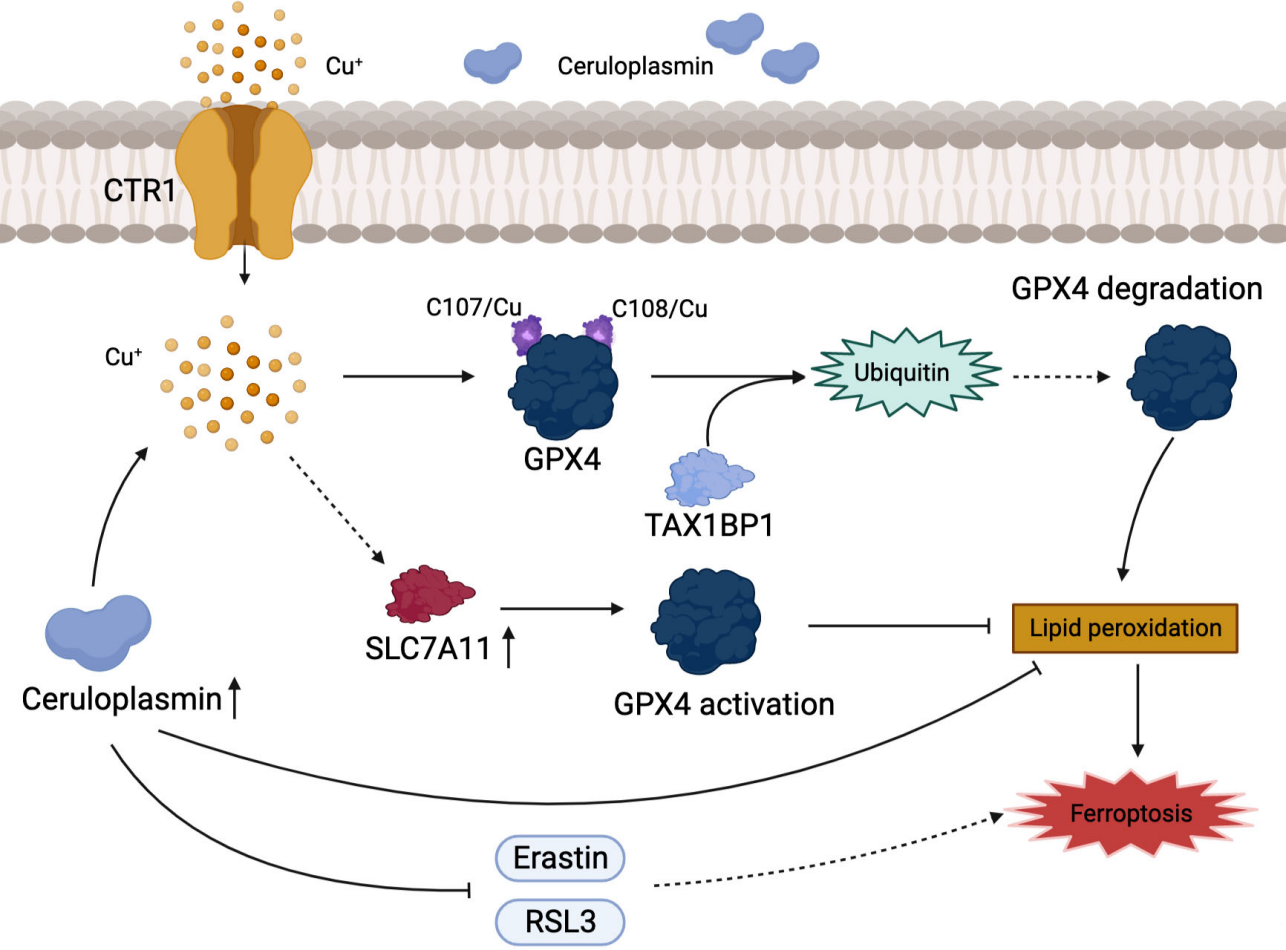
Association of copper metabolism with ferroptosis in HCC. Cu^+^ regulates the level of ferroptosis in HCC by activating GPX4 or promoting its ubiquitination. CP inhibits ferroptosis by directly inhibiting the iron death inducers Erastin and RSL3.

Ferroptosis is a recently identified form of cell death characterized by iron accumulation and lipid peroxidation that has emerged as an effective therapeutic target for tumor suppression. We are well known for the fact that sorafenib can inhibit the progression of HCC by inducing ferroptosis (77). However, copper and iron which are common trace elements in human body, the association between copper metabolism and ferroptosis has been rarely reported. GPX4 is a key gene associated with ferroptosis. Exogenous copper promotes GPX4 ubiquitination by directly binding to GPX4 protein cysteines C107 and C148, accelerating GPX4 aggregate formation, and Tax1-binding protein 1 (TAX1BP1) is involved in the transformation breakdown of GPX4, leading to lipid peroxide accumulation and inducing ferroptosis (78). The plasma ceruloplasmin-ferroportin transport system is an active mode of intracellular iron transport in hepatocytes. Shang et al. (79) discovered that high copper level disrupted Cu-Fe homeostasis and that overexpression of CP inhibited ferroptosis induced by erastin and RSL3 in HCC cells. Despite the fact that ionizing radiation can slow tumor cell development by increasing ferroptosis, which is beneficial for patients with advanced HCC that cannot be surgically removed, the efficacy of radiation is hampered by radioresistance (80, 81). Notably, copper metabolism MURR1 domain 10 (COMMD10) is a critical protein in the regulation of radioresistance in HCC, which causes copper aggregation, thereby upregulating the expression of CP and SLC7A11 target genes, reducing lipid peroxidation levels, and inhibiting ferroptosis in HCC (82). Excessive accumulation of free intracellular copper causes cell death, and a similar result occurs when the intracellular copper content is depleted. Copper depletion-mediated metabolic reprogramming leads to mitochondrial perturbations and significant changes in lipids and lipid-like molecules (including increased levels of arachidonic and epinephrine acids), with high levels of ROS and lipid peroxidation strongly inducing ferroptosis (83). Interestingly, DSF/Cu which is combined with copper is an FDA approved clinical anti-alcoholic drug, and Ren et al. found that DSF/Cu can activate ferroptosis and synergize with sorafenib in the fight against HCC (84). There is a direct link between ferroptosis and copper, with copper acting as a crucial factor in the regulation of ferroptosis (**Figure 3**).

## 4 Anti-tumor therapy to regulate copper homeostasis

With the in-depth study of copper metabolism, the effects of copper on human health are becoming clear. How to safely, effectively and rationally use drugs to regulate copper homeostasis to treat diseases has attracted people’s attention. Previous treatments for inherited copper dysregulation diseases (such as Wilson disease, Menkes disease) provide reference for this. In terms of tumors, there are two main ways to regulate copper homeostasis: one is to chelate copper in tumor cells through copper chelates to inhibit copper proliferation; the other is using copper ionophores to increase the copper in tumor tissue to promote cuproptosis.

### 4.1 Copper chelators

Copper chelators are commonly used to treat Wilson disease patients to lower their elevated copper in the blood. In the subsequent research, it was observed that anti-angiogenic agents can limit tumor growth and that copper restriction can reduce the levels of angiogenic factors to inhibit the angiogenesis of blood vessels (85, 86). Copper chelating agents have aroused people’s interest in the field of cancer. Researches have shown that copper can affect tumor angiogenesis by affecting HIF-1 (87); participat in tumor proliferation through the transcription factor ATOX1 (21), CCO (88) and MEK1 (89); regulate the Mediator of Cell Mobility protein (MEMO) (90), the Secreted Protein Acidic and Rich in Cysteine (SPARC) (91, 92), the copper binding enzymes LOX (93), to influence tumor spreading. Additionally, it has been documented that copper chelation can also promote ubiquitin mediated degradation of the immune checkpoint PD-L1, such that tumor cells cannot protect themselves from antitumor immune responses by overexpressing PD-L1 (56).Therefore, copper chelators may act on tumors through these pathways. At present, the inhibitory effect of copper chelators on tumor angiogenesis has received much attention. A crucial aspect of HCC is its abundant arterial blood supply. It is ideal for treatment with copper chelators due to this property. We believe that copper chelators have a bright future in the treatment of HCC.

**Figure 4:**
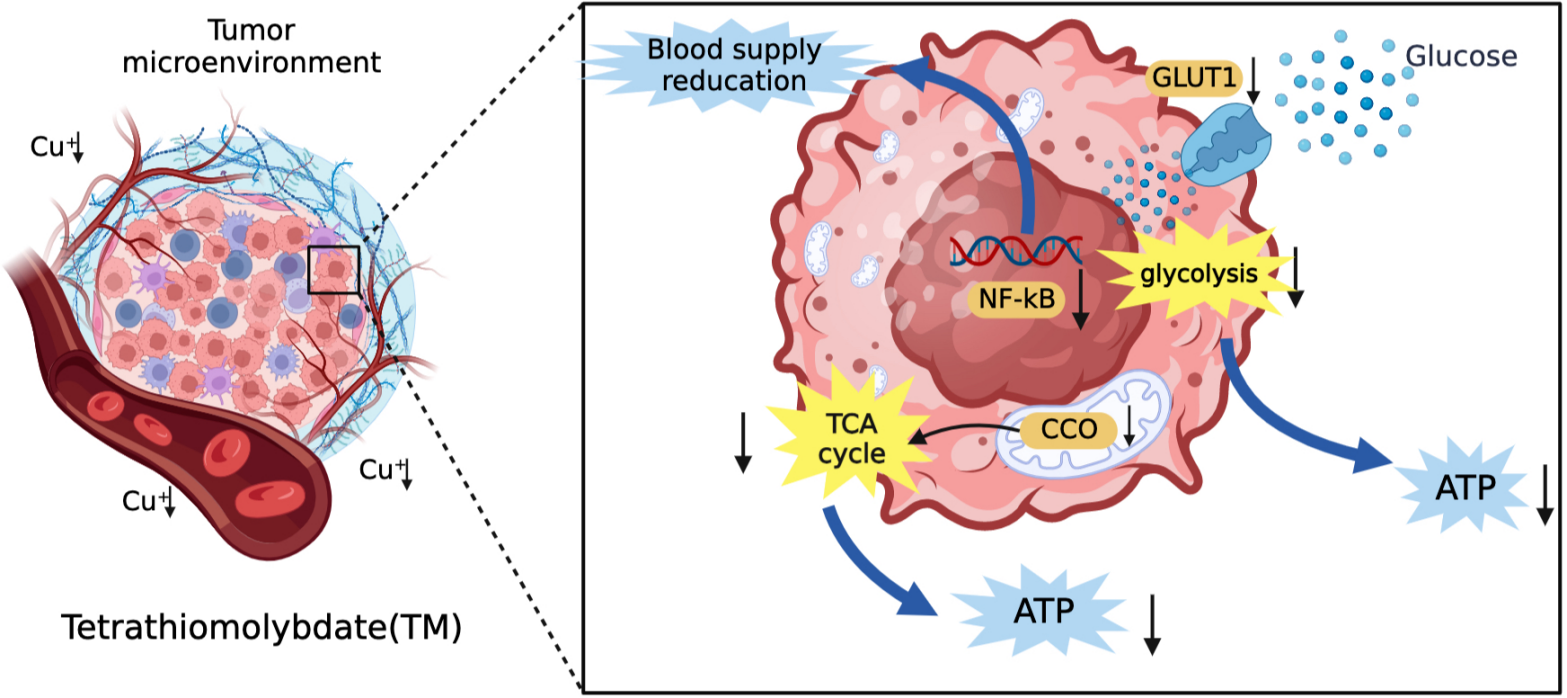
The mechanism of TM for the treatment of HCC. TM can inhibit angiogenesis and reduce vascular invasion of HCC by inhibiting transcription factors (NF-kB). In addition, it can also reduce the vascular invasion of HCC by inhibiting the tricarboxylic acid cycle and reducing the energy supply of tumor tissue by glycolysis.

Common copper chelators include tetrathiomolybdate (TM) and trientine. TM is a selective copper chelator which could deplete copper in tumor cells and reduce its bioavailability. TM can inhibit angiogenesis and reduce blood supply to tumor tissues by supressing transcription factors (NF-kB) and reduce tumor proliferative activity by inhibiting mitochondrial CCO function to reduce ATP production (88, 94). In addition, Davis et al. (95) suggested that TM could reduce the expression of glucose transporter 1 (GLUT1) and other glycolytic genes induced by hypoxia in HCC cells to decrease glucose utilization and limit energy acquisition. This shows that TM can limit the production of ATP in tumor cells through mitochondrial tricarboxylic acid cycle and glycolysis. The mechanism of TM for the treatment of HCC is shown in **Figure 4**. The safety of TM has also been recognized. In a phase II clinical trial, TM was well tolerated (96). Trientine is commonly used in patients who are intolerant to other copper chelators due to severe side effects such as myelosuppression and autoimmune diseases. KADOWAKI et al. (97) suggested that trientine induces tumor apoptosis by activating P38 mitogen-activated protein kinase, which is involved in multiple pathways to inhibit the growth of HCC and other tumors (98–101). A study using trientine in a mouse model of HCC xenotransplantation showed that trientine could limit tumor growth by inhibiting the growth of HCC endothelial cells and blood vessels, and promote the induction of tumor apoptosis (102). It is safer compared to copper chelator D-pen with lower incidence of serious side effects (103). Therefore, we believe that trientine has potential for clinical use in the treatment of HCC.

### 4.2 Copper ionophores

In contrast to copper chelators which deplete copper bioavailability in tumor tissue to inhibit cuproplasia with the aim of achieving antitumor effects, copper ionophores increase the intracellular concentration of copper ions in tumor cells. Excessive intracellular copper ion concentration has cytotoxicity as it can lead to the production of a large amount of ROS (104). Additionally, copper ions directly attach to the lipoylated parts of the tricarboxylic acid (TCA) cycle, causing lipoylated protein to aggregate and Fe-S cluster protein to disappear (69). This causes protein toxicity stress, which kills or restricts tumor growth.

**Figure 5:**
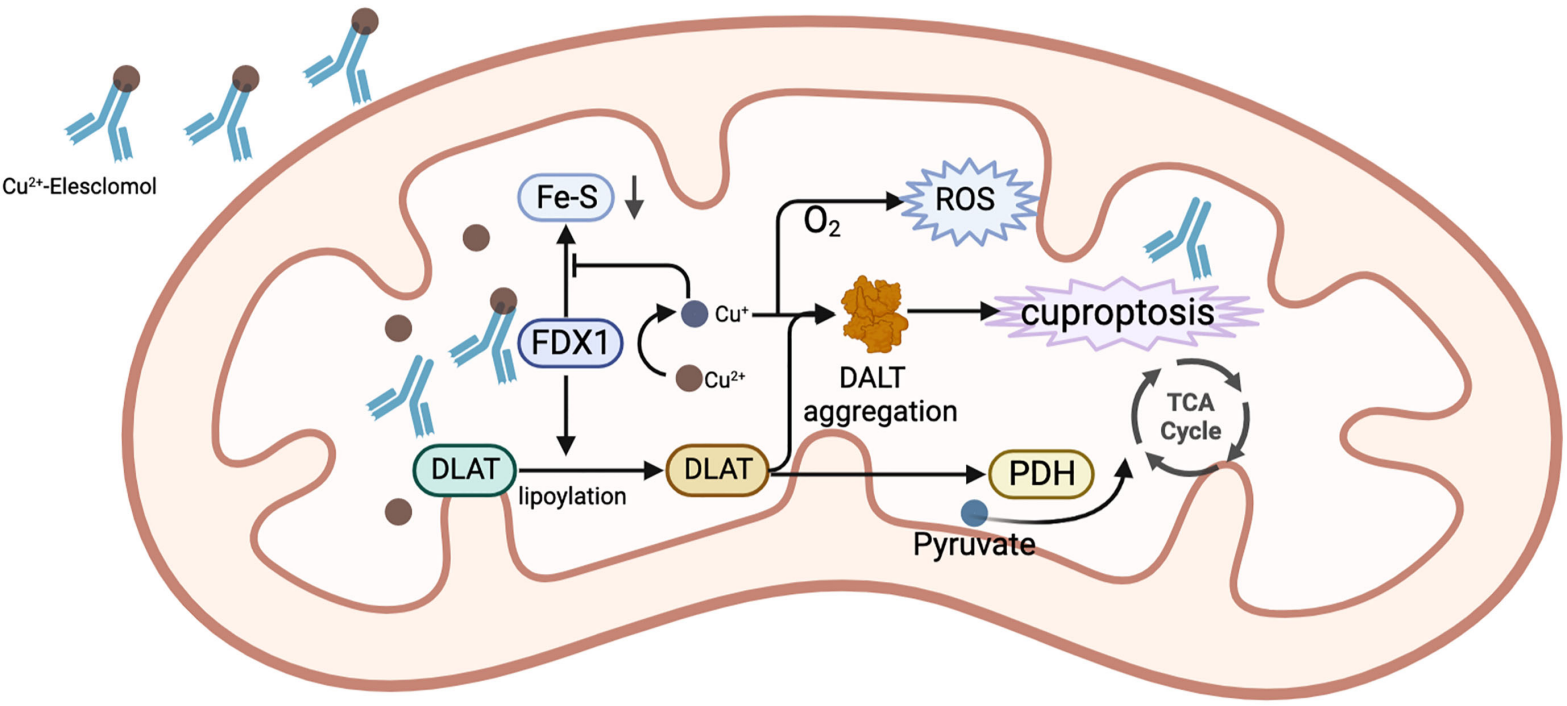
The mechanism of ES for the treatment of HCC. In mitochondria, the complex of ES and Cu^2+^ binds to FDX1, while Cu^2+^is reduced to Cu^+^ by FDX1. Cu^+^ combines with O_2_ to generate ROS. FDX1 also promotes the lipoylation of DLAT. The reduced Cu^+^ binds to lipoylated DLAT to promote its oligomerization, ultimately causing the occurrence of cuproptosis. Additionally, Cu^+^ prevents FDX1 from stimulating the synthesis of Fe-S.

Currently, common copper ionophores are elesclomol(ES) and disulfiram(DSF). ES is an injectable, small molecule drug that binds to copper ions in the blood. Tumor cells can effectively absorb this complex. When Cu^2+^ of this complex enters the mitochondria of cells, it is reduced to Cu^+^ by mitochondrial protein FDX1. Cu^+^ will react with molecular oxygen to produce superoxide, which will be disproportionated to generate H_2_O_2_. H_2_O_2_ can further react with Cu^+^ to produce more destructive and highly reactive hydroxyl radicals, destroying mitochondria, and restricting the division and proliferation of tumor cells (105). Moreover, ES can also downregulate GSH (106), which is an important component of the intracellular antioxidant system and contributes to the clearance of intracellular ROS (107). Without a doubt, its decrease leads to the rise in intracellular ROS. Additionally, Tsvetkov et al. (65, 69) argued that ES has anti-tumor properties that go beyond merely its capacity to generate ROS in tumor cells. ES was discovered to be able to bind to FDX1, which results in the the aggregation of lipoylated mitochondrial enzymes and a loss of Fe–S proteins, leading to cuprotosis. Since this process requires the involvement of oxygen molecules, this drug is mainly suitable for cancers that are energized by oxidative phosphorylation of mitochondria. Under hypoxic conditions, the energy metabolism of tumors is mainly generated through glycolysis in the cytoplasm rather than the mitochondria, which is often accompanied by increased levels of the lactate dehydrogenase (LDH). At this time, the activity of ES is low. HCC cells have been reported to have a characteristic of significantly increased mitochondrial metabolism (108). This provides a theoretical basis for the use of ES in the treatment of HCC. Another study on carboplatin-resistant HCC cells also showed this feature (109). In terms of safety, ES has few reported side effects in humans and is well tolerated by patients. It was found that while ES was present at concentrations that significantly inhibited tumor cells, it did not enrich for copper ions in human peripheral blood mononuclear cells (105). This also suggests that the risk of side effects from taking this drug is low. The mechanism of elesclomol for the treatment of HCC is shown in **Figure 5**.

DSF was used to treat alcohol dependence by inhibiting acetaldehyde dehydrogenase (ALDH). In the 1970s, researchers began seeking clinical evidence of the anti-cancer effects of DSF (110). DSF has anticancer action that is reliant on metal ions and promotes apoptosis while decreasing angiogenesis. In acidic conditions, DSF would be reduced to diethyl dithiocarbamate (DDTC), which may then be coupled with Cu^2+^ to create DDTC-Cu^2+^ complexes. There are a number of possible mechanisms of action for DSF and DDTC-Cu^2+^, including the generation of ROS (111), obstruction of the ubiquitin pathway (112), activation of the MAPK pathway (113), and irreversible inhibition of ALDH (114). In addition, a study by Li et al. (115) demonstrated that DSF in combination with copper could effectively inhibit the metastasis of HCC, suggesting that DSF in combination with copper could restrain the process of epithelial-mesenchymal transition (EMT) and the metastasis of HCC by limiting NF-kB and TGF-Β signaling. Concerning security, DSF has been approved by FDA for application, and its safety is generally acceptable. Current trial phase and validated targets information on copper chelators and copper ionophore for HCC are shown in **Table 2**.

**Table 2:** Current trial phase and validated targets information on copper chelators and copper ionophore for HCC.

Type	Copper regulator	Abbreviation	Molecular formula	Trial Phase	Related targets	Refs
chelator	Tetrathiomolybdate	TM	MoS4	Animal model	NF-kB	(86, 116)
				Cell and animal models	MEK1/2	(48, 117)
				Cell model	IL-1	(118, 119)
				Cell model	FGF1	(118, 119)
				Animal model, Phase II trial	VEGF	(120–122)
				Cell and animal models, Phase II trial	LOX	(116, 123)
				Animal model	CCO	(88)
				Phase II trial	IL-6	(121, 122)
				Phase II trial	IL-8	(121, 122)
				Phase II trial	bFGF	(121)
chelator	Trientine	TETA	C6H18N4	Cell model	IL-8	(124)
				Animal model	CD31	(102)
				Cell model	P38 MAPK	(97)
chelator	D-penicillamine	DPA	C5H11NO2S	Animal model	LOX	(125)
chelator	Choline tetrathiomolybdate	ATN-224	[(CH3)3NCH2CH2OH]2[MoS4]	Cell model	SOD1	(126)
chelator	Tetraethylenepentamine pentahydrochloride	TEPA	C8H23N5·5HCl	Cell model	PD-L1	(56)
				Cell model	HIF-1	(87)
ionophore	Elesclomol	ES	C19H20N4O2S2	Cell model	GSH	(106)
				Cell and animal models	FDX1	(65, 69)
ionophore	Disulfiram	DSF	C10H20N2S4	Cell model	ALDH	(127)
				Cell model	ubiquitin protein pathway	(112)
				Animal model	NF-kB	(115, 128)
				Cell model	P38 MAPK	(113)
ionophore	Clioquinol	CQ	C9H5ClINO	Cell model	Proteasome	(129)

### 4.3 Copper related imaging

The demand of tumor cells for copper ions and the transport of copper ions by CTR1 on the surface cause high copper concentration in tumor tissues. This makes copper complexes have the potential to act as tracers. The radioisotope ^64^Cu has been used for *in vivo* tumor imaging and therapy. ^64^Cu has a half-life of 12.7 hours and is suitable for imaging small molecules as well as large molecules such as antibodies and peptides. Its relatively short half-life does not add a radiation burden to patients after imaging studies, which is a advantage that can be applied to positron emission tomography (PET) imaging. Currently, ^64^CuCl2 has performed well in PET imaging of HCC in animal models (130).

In addition, copper-based nanoparticles have been applied in both photodynamic therapy PDT and photothermal therapy PTT. Huang et al. (131) used copper-cysteamine nanoparticles as photosensitizers for HCC and achieved good therapeutic results. It is notable that the homeostasis of copper ions in the human body is essential, and either its deficiency or its excess can cause human diseases. The long-term use of copper binding compounds, including copper chelators and copper ionophores, may disturb the homeostasis of essential metals, thus resulting in severe side effects. Although copper conjugates exhibit certain selectivity toward tumor cells, their therapeutic window still needs to be enlarged for safer applications. There is a need to develop more rational strategies and new therapeutic modalities to increase targeting to tumor cells, improve efficacy against tumors, as well as mitigate side effects.

## 5 Conclusion

With the development of cross-cutting disciplines, the pathways of copper metabolism involved in human activities have been elucidated. Copper is absorbed in the small intestine and stored by vital organs such as the liver. Copper is essential for the regulation of the physiological functions of the liver. Excess copper would be excreted via the biliary tract when copper levels in the body are high. However, the state of cuproplasia is commonly seen in the microenvironment of HCC. Copper overload accelerates the progression of HCC through immunosuppression, vascular invasion, cuproptosis and ferroptosis. Current therapies on copper metabolism in HCC include copper chelators, copper ionophores, etc., all of which have a good safety profile. Currently, copper metabolism research is in a preliminary stage and this therapy is not explicitly recommended in the guidelines despite clear therapeutic effects, so further clinical studies are still urgently needed.

## Author contributions

CZ, TL and JY gathered information and designed the review. PS and RJ drew the pictures. LY and WZ critically revised the manuscript. All authors contributed to the article and approved the submitted version.
